# Retroperitoneal fibrosis associated with propranolol: a case report; is corticosteroid administration necessary after ureterolysis?

**DOI:** 10.12861/jrip.2013.22

**Published:** 2013-06-01

**Authors:** Majid Shirani, Azadeh Davoudian, Abolghasem Sharifi

**Affiliations:** ^1^Department of Urology, Shahrekord University of Medical Sciences, Shahrekord, Iran; ^2^Deputy for Research Affairs, Shahrekord University of Medical Sciences, Shahrekord, Iran

**Keywords:** Retroperitoneal fibrosis, Corticosteroids, Ureterolysis

## Abstract

**Introduction:** Retroperitoneal fibrosis is a rare disease. It can be primary (Ormond’s disease) or secondary to inflammation, malignancy or some drugs. Beta-adrenergic blockers including propranolol can cause the retroperitoneal fibrosis disease.

**Case:** A 44-year-old woman who was taking propranolol for 13 years came to our center with complaints of oliguria and uremia symptoms (malaise, nausea and vomiting). After some investigations, it was found that the disease was retroperitoneal fibrosis. In the first step, she was treated with corticosteroids and then because of inadequate response, bilateral ureterolysis was performed. Then, an additional course of corticosteroid therapy was required after surgery.

** Conclusion:** Retroperitoneal fibrosis is an unknown cause disease that can involve ureters and can cause obstructive symptoms. The imaging procedure of choice for diagnosis is abdominal CT scanning with oral and intravenous contrast agents. Corticosteroids are the first option for treatment, however, if they are not effective and in case of severe obstruction, ureterolysis can be performed. Beta- adrenergic blocker drugs that are widely used in heart diseases can be a cause of retroperitoneal fibrosis.

**Implication for health policy/practice/research/medical education:**

Retroperitoneal fibrosis is an unknown cause disease that can involve ureters and cause obstructive symptoms. The useful imaging method for diagnosis retroperitoneal fibrosis is abdominal CT scanning with oral and intravenous contrast agents. Corticosteroids are the first option for treatment. However, if they were not effective and in cases of severe obstruction, ureterolysis can be performed. Beta-adrenergic blocker drugs that are widely used in heart diseases can be a cause of retroperitoneal fibrosis.

## Introduction

Retroperitoneal fibrosis is characterized by the proliferation of fibrous tissue in the retroperitoneal space. It may occur as a primary idiopathic retroperitoneal fibrosis (Ormond’s disease) or as a secondary disease in response to inflammation, malignancy, drugs, surgery, infection or radiation therapy (1,2).

Retroperitoneal fibrosis is an uncommon condition with an estimated incidence of 1.38 cases per 100,000 people (1-3). Its peak prevalence is observed in the 40-60 years of age and the ratio of male to female is reported to be 2 to 1 (4,5). The probability of an allergic or autoimmune mechanism has been put forward for this disease. Fibrosis usually occurs around the abdominal aorta and iliac vessels below the renal arteries (2).

The main characteristic and also main complication of this disease is asymptomatic and progressive involvement of ureters (2). Different drugs that have been noted to cause idiopathic fibrosis include: ergotamine, propranolol, methysergid, hydralazin, methyldopa, metoprolol and some analgesics.

## Case

The patient was a 44-year-old woman, a known case hypothyroidism who has been under treatment with levothyroxine, propranolol, and clonazepam since 13 years ago. The patient was referred to the emergency ward, with uremia symptoms (weakness, nausea and vomiting) and oliguria. In the preliminary management, the patient was placed under hemodialysis treatment due to high blood urea nitrogen and creatinine (BUN=112 mg/dl, Cr=10 mg/dl), electrolyte imbalance (K=6.5 mg/dl) and uremia symptoms. Patient stated a history of vague abdominal pain since the last year. In paraclinical evaluations, conducted during hospitalization, patient suffered with chronic anemia (Hemoglobin=9.8 g/dl) as well, bilateral fullness of pelvicalyceal system was reported on ultrasonography of the kidneys. In the next phase, spiral abdominopelvic CT-scan without intravenous contrast was performed that showed bilateral mild hydronephrosis. Moreover, a dense fibrotic plaque was observed, surrounding the central abdominal vessels. JJ stents were placed for the patient in the ureters on both sides, since, it was suspected inflammatory process surrounding the ureters and considered the probability of the two-sided obstructive factor led to increased creatinine and oliguria. After JJ stent insertion, diuresis was established and BUN/Cr decreased. The patient did not require dialysis anymore. The medial deviation was clearly observed in both ureters after placing stents on KUB. Propranolol was stopped; the patient was left out with prescription of corticosteroid drugs (prednisolone 50 mg daily). After 5 weeks, patient’s stents were removed. However, anuria occurred and creatinine increased. Stents were placed in both ureters and diuresis established again. After 3 weeks, despite corticosteroid therapy and ureteral catheterization oliguria and rising in BUN/Cr occurred. Thus, the patient was candidate for surgery. Ureterolysis was done and ureters were placed in peritoneum.

Samples obtained from the fibrotic tissues and were sent to the pathology laboratory, however, no evidence of malignancy was found. After the fourth week, JJ catheters were removed, but the patient showed a gradual increase in creatinine level after about a week (1.2 to 3.1). Two options were proposed for the patient’s management. One of them was inserting ureteral stents again or beginning treatment with corticosteroid that the second option was adopted. Serum creatinine was declined to 1.5 mg/dl and remained constant after consumption of prednisolone 50 mg/day. After one month of treatment, drug tapered gradually and was interrupted after 4 months. After 6 months of follow- up, patient had not any complaint and serum BUN/Cr levels remained constant.

## Discussion

Retroperitoneal fibrosis is a rare cause of renal failure with a silent clinical course. Early inflammatory reaction involves T cells (T helper), plasma cells and macrophages significantly. Then, these cells are replaced with the collagen synthesizing fibroblasts subsequently (1). The retroperitoneal fibrosis process begins right from below the renal artery. Fibrosis develops gradually and surrounds ureters, inferior vena cava, aorta and mesenteric vessels or sympathetic nerves. Bilateral involvement was reported in 67% of cases. Retroperitoneal fibrosis can also be secondary to a variety of inflammatory situations or be allergic reactions to medications. These inflammatory conditions include abdominal aortic aneurysm, pancreatitis, histoplasmosis, tuberculosis and actinomycosis. Retroperitoneal fibrosis can also be related to a variety of malignancies including prostate, pancreas and stomach cancers. It can be associated with non-Hodgkin lymphoma, stromal and carcinoid tumors, too. Retroperitoneal fibrosis has been seen with some autoimmune diseases, including ankylosing spondylitis, systemic lupus erythematous, Wegener’s granulomatosis, and polyarteritis nodosa (1,6). The primary symptoms include a dull and non-localized pain in the flanks, back and abdomen (6,7). The pain is usually associated with weakness, anorexia, weight loss, fever, back pain and high blood pressure. In the next stage of the disease, symptoms occur due to obstructive uropathy and azotemia (8). Microscopic hematuria and anuria may occur (9,10).

Early diagnosis of retroperitoneal fibrosis is possible, using imaging studies. The classic diagnostic triad in excretory urography include hydroureteronephrosis in proximal parts of the ureters, medial deviation of the ureters, and the evidence of external pressure on them (4,7,8). A long segment of ureters usually involves and absence of peristalsis due to fibrosis in some cases is led to pipe stem appearance (11). Laboratory evaluation may show an increase of BUN and creatinine or erythrocyte sedimentation rate (ESR). At present, abdominopelvic CT-scan with oral and intravascular contrast agents is the useful imaging method for diagnosis and shows the size and extent of the fibrotic process generally. If renal function is compromised, MRI can be a helpful imaging procedure (1). Although some cases of spontaneous regression have been reported, treatment of this disease is usually surgical. A course of corticosteroid therapy may be tried first and obtains favorite results in some cases. If the patient fails to respond to corticosteroid or in cases with sever obstruction and increase creatinine level, such as in our patient, it will be necessary to separate ureters from fibrotic plaque with surgery. After releasing the ureters, they are wrapped with omentum or brought to an intraperitoneal location to prevent their recurrence (11). Demko and colleagues reported a patient with an increase in blood pressure and the progressive renal insufficiency. After confirming the diagnosis of retroperitoneal fibrosis by radiographic studies, the patient underwent ureterolysis surgery and this caused a creatinine decrease (3). There was a difference in our patient in spite of ureterolysis treatment. There was a gradual increase in creatinine level that led to starting corticosteroid therapy again. In another case, reported by Skehan *et al*., a patient with retroperitoneal fibrosis due to metoprolol was treated by ureterolysis (12). The success rate of ureterolysis operation has been reported to be between 66-100%. Most of physicians discontinue medical treatment after surgery and a few prescribe medical therapies with corticosteroids or other immunosuppressive drugs. In a study performed by Jung *et al*. on 27 patients with fibrosis of the retroperitoneum, the use of immune suppressive therapy like glucocorticoid and azathioprine reduced acute phase proteins and decreased the fibrotic mass size in most patients (2). There is no identical opinion about medical treatment and the dose and duration of treatment yet (13). After removal of stents in this patient, we had to administer corticosteroid due to a creatinine rise, and obtained a suitable response. This finding reveals that, it is necessary to care patient after surgery carefully. In cases of returning symptoms, at least, the use of corticosteroids can help in some patients.

Clinical use of beta-blockers is widespread. Beneficial effects of this drug on cardiovascular system especially at hypertension, angina and arrhythmia is very important and popular. The treatment of chronic congestive heart failure is a new application for this drug (14). Since the beta blocker medications use in high level to treat high blood pressure and angina, some cases of idiopathic fibrosis inevitably be attributed to the use of these drugs (12).

## Conclusion

Retroperitoneal fibrosis is an unknown cause disease that can involve, ureters, and can cause obstructive symptoms. The imaging procedure of choice for diagnosis it is abdominal CT scanning with oral and intravenous contrast agents. Corticosteroids are the first option for treatment, if they could not yield optimal response, and in cases of severe obstruction, ureterolysis can be performed. Beta- adrenergic blocker drugs that are widely used in heart diseases can be a cause of retroperitoneal fibrosis.

## Authors’ contributions

MSh and AD prepared the primary draft. ASh wrote some parts of the manuscript and edited the paper.

## Conflict of interests

The author declared no competing interests.

## Ethical considerations

Ethical issues (including plagiarism, informed consent, misconduct, double publication and redundancy) have been completely observed by the authors.

## Funding/Support

None declared.
